# Sebaceous filaments of the nose treated with a 1726-nm diode laser: A case report and description of the “Lunar crater sign”

**DOI:** 10.1016/j.jdcr.2026.01.001

**Published:** 2026-01-10

**Authors:** Abdulaziz Almufadhi, Mohammed Alosaimi

**Affiliations:** a Division of Dermatology, King Abdulaziz Medical City, Ministry of National Guard Health Affairs, Riyadh; b King Saud bin Abdulaziz University for Health Sciences, College of Medicine, Riyadh

**Keywords:** dermoscopy, diode laser, laser therapy, sebaceous filaments, selective photothermolysis

## Case presentation

A 30-year-old man with no significant medical history, Fitzpatrick skin phototype IV, presented with prominent nasal sebaceous filaments for more than 5 years.

The patient was treated with topical retinoid (tretinoin 0.05 cream once daily) for 6 months with minimal improvement.

**Fig 1:**
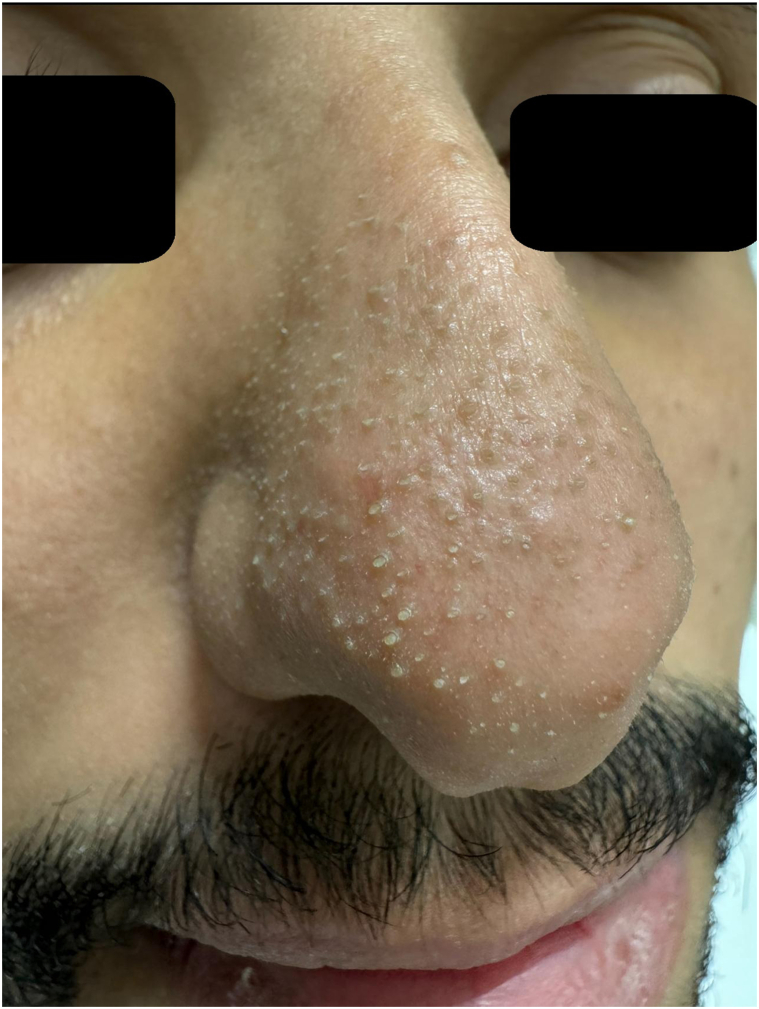
Baseline clinical photo of the nose showing prominent sebaceous filaments.

**Fig 2:**
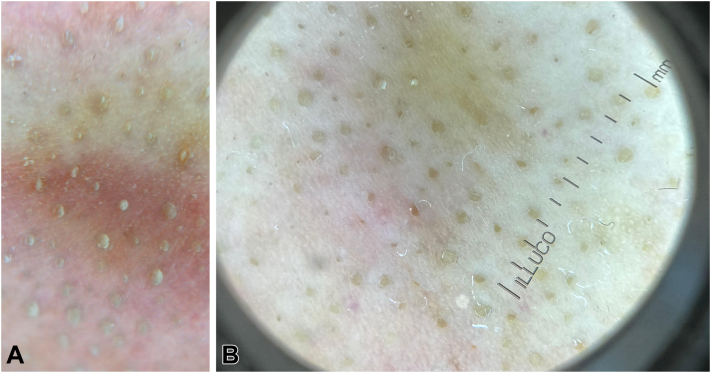
Dermoscopy (ILLUCO IDS-1100C, × 20): **A,** baseline showing *yellowish* follicular plugs; **B,** one month post-treatment showing *brown-to-tan* crateriform globules “Lunar crater sign.”

Dermoscopy (polarized contact, ILLUCO IDS-1100C, × 20) demonstrated uniform yellowish plugs consistent with sebaceous filaments.^1^ (Fig 1 and Fig 2, *A*).

## Intervention

Device: AviClear (CUTERA), 1726-nm diode; integrated contact cooling.

Settings: Single session; 3.0-mm spot; fluence 18 J/cm^2^; 20 pulses; PRILA 5% (lidocaine/prilocaine cream) for 30 minutes before the procedure.

Immediate response included vaporization of the sebaceous material, without purpura or blistering.

Aftercare: Broad-spectrum sunscreen (sun protection factor 50).

## Outcomes

**Fig 3:**
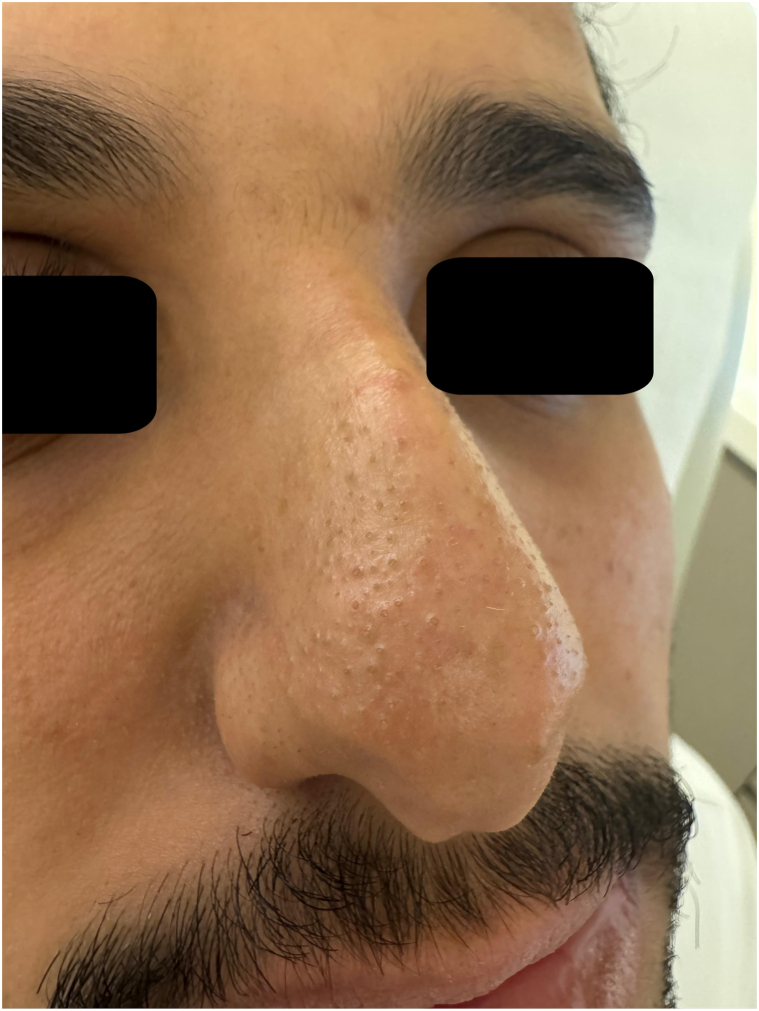
Post-treatment clinical photo of the nose at 4 weeks.

At 4 weeks, clinical improvement was evident (Fig 3).

Dermoscopy showed brown-to-tan crateriform globules, proposed as the “Lunar crater sign” due to resemblance to lunar craters (Fig 2, *B*).

Adverse effects: None reported by the patient, with no scarring or postinflammatory hyperpigmentation.

## Discussion

Sebaceous filaments are physiologic accumulations of sebum and corneocytes in the follicular infundibulum and appear as uniform off-white/yellowish plugs on dermoscopy.^1^ They should be differentiated from trichostasis spinulosa and pityriasis folliculorum; prior reports describe improvement with topical keratolytics/retinoids.^2^

Light-based modalities such as intense pulsed light and the 1450-nm diode laser have previously been used to target sebaceous-gland activity, mainly in acne vulgaris and seborrhea.^3^ These devices achieve photothermal damage of sebaceous glands with transient sebum reduction. However, no prior reports describe their use for isolated sebaceous filaments. The 1726-nm wavelength offers greater lipid absorption and thus more selective sebaceous-gland photothermolysis.^4^

The 1726-nm wavelength targets sebum with higher absorption than water, enabling selective sebaceous-gland photothermolysis.^4^^,^^5^

Prospective acne cohorts demonstrate sebaceous-gland miniaturization and progressive lesion reduction durable to at least 26 weeks, with mostly transient erythema/edema and rare significant adverse effects.^5^, ^6^, ^7^

To our knowledge, 1726-nm treatment of isolated sebaceous filaments has not been specifically reported; this case suggests feasibility and motivates controlled evaluation.

### Limitations

Single patient, short follow-up, no histology, and photography/lighting confounders.

## Conflicts of interest

None disclosed.
